# Ferulic acid mitigates 3-Nitropropionic acid-induced Huntington’s disease via modulation of Nrf2/HO-1, TLR4/NF-κB, and SIRT1/p53 signaling pathways

**DOI:** 10.3389/fphar.2025.1678724

**Published:** 2025-10-01

**Authors:** Mohamed A. Abdelgawad, Abdallah M. Gendy, Sameh S. Zaghlool, Wessam H. Elesawy, Mai F. Ragab, Mohamed I. Kotb El-Sayed, Alaadin E. El-Haddad, Hussein S. Mohamed, Izzeddin Alsalahat, Marwa A. Essa

**Affiliations:** ^1^ Department of Pharmaceutical Chemistry, College of Pharmacy, Jouf University, Sakaka, Saudi Arabia; ^2^ Pharmacology and Toxicology Department, Faculty of Pharmacy, October 6 University, Giza, Egypt; ^3^ Pharmacology and Toxicology Department, Faculty of Pharmacy, Modern University for Technology and Information (MTI), Cairo, Egypt; ^4^ Department of Pharmacology and Toxicology, College of Pharmaceutical Sciences and Drug Manufacturing, Misr University for Science and Technology, Giza, Egypt; ^5^ Pharmacology Department, School of Life and Medical Sciences, University of Hertfordshire Hosted by Global Academic Foundation, Cairo, Egypt; ^6^ Biochemistry and Molecular Biology Department, Faculty of Pharmacy, Helwan University, Helwan, Egypt; ^7^ Pharmacognosy Department, Faculty of Pharmacy, October 6 University, Giza, Egypt; ^8^ Chemistry of Medicinal and Aromatic Plants Department, Research Institute of Medicinal and Aromatic Plants (RIMAP), Beni-Suef University, Beni-Suef, Egypt; ^9^ International Study Centre, Cardiff University, Cardiff, United Kingdom; ^10^ Biochemistry Department, Faculty of Pharmacy, October 6 University, Giza, Egypt

**Keywords:** 3-Nitropropionic acid, ferulic acid, SIRT1, Nrf2, neuroinflammation, TLR4

## Abstract

**Background:**

Ferulic acid (FA) is a natural phenolic compound that has demonstrated effectiveness against Huntington’s disease (HD). However, its exact mechanism remains unclear. Therefore, the current study aims to investigate FA’s potential mechanism of action against 3-nitropropionic acid (3NP)-induced HD.

**Methods:**

Adult male Wistar albino rats were administered FA orally (100 mg/kg) for 3 weeks, and 3NP (10 mg/kg) was intraperitoneally administered during the last 2 weeks to induce HD. Behavioral performance was assessed using the open field and hanging wire tests. Striatal tissue was analyzed using ELISA, qRT-PCR, Western blotting, histopathology, and immunohistochemistry.

**Results:**

Administration of 3NP led to weight loss, neurobehavioral deficits, oxidative damage, apoptotic cell death, and neuroinflammation. FA treatment mitigated these pathological changes by activating Nrf2/HO-1 signaling, a critical player in cellular redox balance. This beneficial effect was mirrored in restoring TAC levels and suppressing MDA. Moreover, FA suppressed TLR4/NF-κB inflammatory signaling, thereby reducing TNF-α and IL-1β levels. In addition, the anti-apoptotic properties of FA were confirmed by modulating SIRT1/p53 signaling, leading to Bcl-2 enhancement and caspase-3 downsizing. Furthermore, FA enhanced neuronal survival and plasticity confirmed by neurotrophic BDNF elevation. Histopathological and immunohistochemical analyses confirmed improved neuronal survival and reduced gliosis following FA treatment.

**Conclusion:**

The current research demonstrates that FA exhibits potent neuroprotective effects in experimental HD by modifying Nrf2/HO-1, TLR4/NF-κB, and SIRT1/p53 signaling pathways. These findings provide new mechanistic insights into FA’s potential role in managing HD.

## 1 Introduction

Huntington’s disease (HD) was initially defined as a convulsive condition, while its formal description as hereditary chorea by George Huntington was provided in 1872 (Gonzalez-Alegre and Afifi, 2006; Túnez et al., 2010) HD is an autosomal dominant neurodegenerative disorder distinguished by cognitive impairment, involuntary movements, and psychiatric symptoms (Chen-Plotkin et al., 2006; Ayala-Peña, 2013). The condition is primarily characterized by progressive damage to the striatum within the basal ganglia (Ayala-Peña, 2013; Túnez et al., 2010). Although the exact mechanisms behind neuronal degeneration in HD remain uncertain, several pathological factors have been implicated, including oxidative stress, disrupted energy, persistent stimulation of astrocytes and microglia, and excessive pro-inflammatory cytokines contribute to the disease process (Palpagama et al., 2019; Paul and Snyder, 2019).

3-Nitropropionic acid (3NP) is a naturally occurring mycotoxin known for its ability to induce HD pathogenesis in experimental animals by inhibiting succinate dehydrogenase enzyme and disrupting mitochondrial energy production (Túnez et al., 2010). This disruption leads to a cascade of harmful events including reduced antioxidant defense mechanisms and the generation of reactive oxygen species generation (Bono-Yagüe et al., 2020). Additionally, 3NP triggers inflammatory responses as evidenced by elevated pro-inflammatory cytokines including intelukin-1 beta (IL-1β) and tumor necrosis factor-alpha (TNF-α) (Valadão et al., 2020). Moreover, it alters glial responses by enhancing glial fibrillary acidic protein (GFAP) expression and activating microglia (Mustafa et al., 2021; Shawki et al., 2021). Furthermore, it promotes caspase-mediated apoptotic initiation, contributing to the neurodegenerative process associated with HD (Goyal et al., 2024).

Silent information regulator 1 (SIRT1) plays a critical protective role in HD (Duan, 2013). SIRT1 activates nuclear factor erythroid 2-related factor 2 (Nrf2)/heme oxygenase-1 (HO-1) antioxidative signaling. Consequently, SIRT1 inhibition is associated with neuroinflammation, oxidative stress, and apoptosis (Huang et al., 2015; Sethi et al., 2025).

In addition, SIRT1 activation can suppress nuclear factor kappa-light-chain-enhancer of activated B cells (NF-κB) signaling and reduce its downstream effects. Moreover, SIRT1 is considered a promising therapeutic target due to its ability to inhibit p53 activity, which is implicated in the apoptotic progression of neurodegenerative diseases (Razick et al., 2023).

Taken together, the SIRT1/Nrf2/NF-κB/p53 axis represents an interconnected signaling network that simultaneously governs oxidative balance, inflammatory responses, and apoptosis in HD. Thus, targeting this axis provides a mechanistic rationale to investigate how ferulic acid (FA) orchestrates multi-faceted neuroprotective effects.

FA is a widely distributed natural bioactive phenolic acid that belongs to the hydroxycinnamic group. FA possesses various biological activities, particularly in vascular endothelial injury, apoptosis, oxidative stress, inflammation, and fibrosis (Li et al., 2021). Notably, previous research has documented the protective effect of FA against different neurological diseases including depression, Alzheimer’s disease, cerebral ischemia-reperfusion injury, epilepsy, and Parkinson’s disease (Thapliyal et al., 2021). Moreover, Denny Joseph (2014) documented the protective effect of FA against 3NP-induced neurotoxicity, however, the underlying mechanisms remain incompletely investigated, which was the goal of the present study. The present study advances this field by demonstrating for the first time that FA can potentially modulate the Nrf2/HO-1, TLR4/NF-κB, and SIRT1/p53 signaling pathways.

## 2 Materials and methods

### 2.1 Drugs and chemicals

The drug (FA, Y0001013) and the toxicant (3NP, N5636) were purchased from Sigma-Aldrich (St. Louis, MO, United States). Both compounds were dissolved in normal saline daily (3NP pH was adjusted to 7.4 using NaOH).

### 2.2 Animals

Adult male Wistar albino rats (210–240 g) were procured from VACSERA (Helwan, Egypt) and acclimatized for 10 days in the animal facility of October 6 University’s Faculty of Pharmacy (O6U), under controlled conditions of ventilation, temperature, light, water, and diet. The experimental protocol adhered to NIH standards and was approved by the O6U Research Ethics Committee (Approval No: PRE-Ph-2411001).

### 2.3 Experimental design

The experiment lasts for 21 days (Danduga et al., 2018). Rats were randomly assigned to four groups (19 rats per group) using a computer-generated random number sequence as follows:

Normal group: Rats received normal saline (the vehicle) for 21 days.

FA group: Rats were given FA (100 mg/kg, p.o.) for 21 days.

3NP group: Rats were injected with 3NP (10 mg/kg, i.p.) from day 8 to day 21 of the experiment.

**FIGURE 1:**
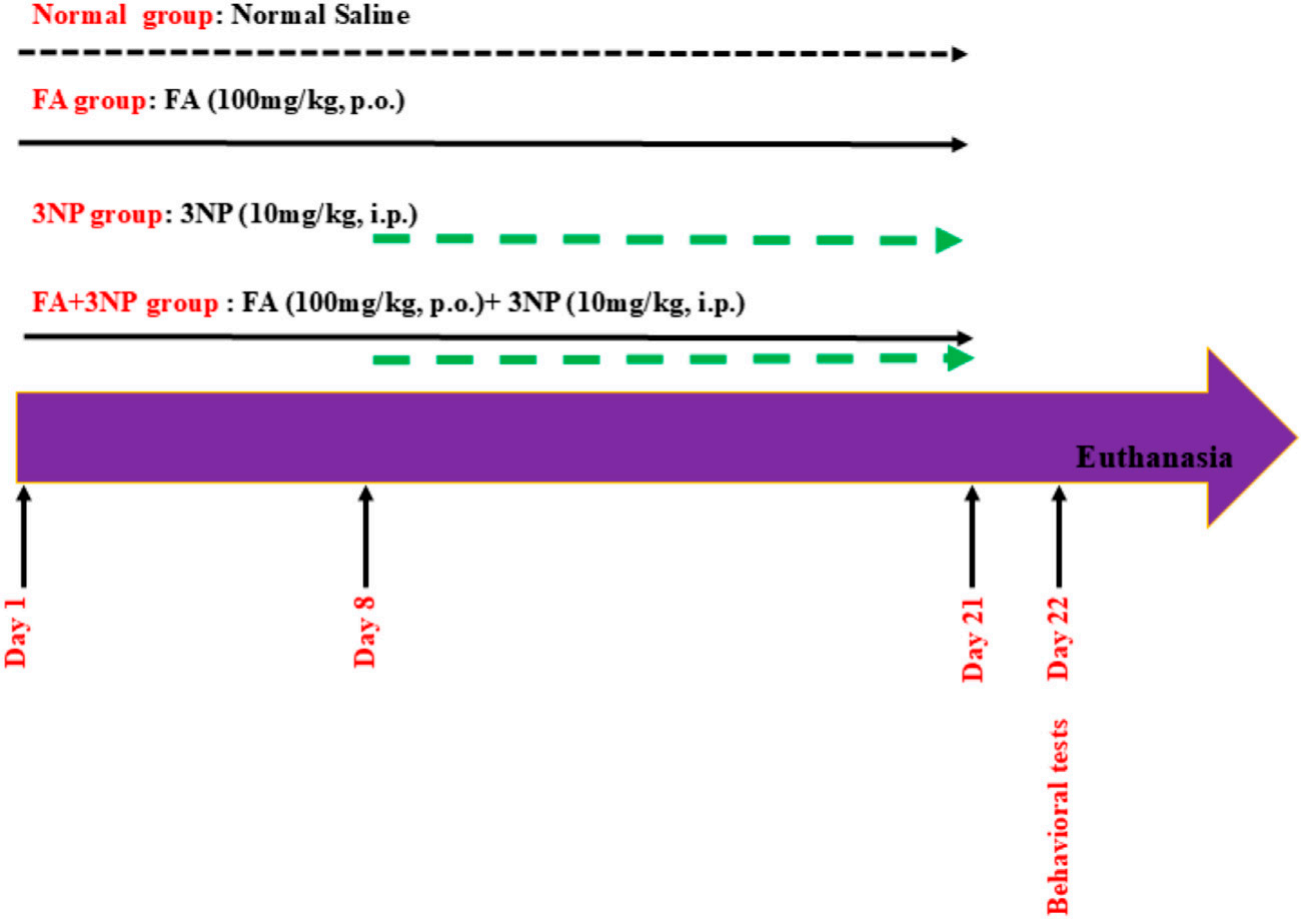
Schematic presentation of experimental design.

FA+3NP group: Rats received FA (100 mg/kg, p.o.) for 21 days (Zhang et al., 2023), administered 1 hour prior to 3NP injections, as previously described (Denny Joseph, 2014), with 3NP (10 mg/kg, i.p.) given only during the last 2 weeks (see Figure 1).

After completing the behavioral experiments on day 22, the animals’ weight was determined then the animals were euthanized by cervical dislocation under thiopental anesthesia (50 mg/kg; i.p.; Cat. No. T1019, Sigma-Aldrich, St. Louis, MO, United States) (Isik et al., 2025) and subdivided into four subsets. Three striatal subsets were stored at −80 °C for biochemical and ELISA assay (n = 6), qRT-PCR (quantitative Reverse Transcription Polymerase Chain Reaction, n = 6), and Western blot (n = 3). The remaining subset (n = 4) was fixed in 10% formalin for subsequent histopathological/immunohistochemical analyses. During the data collection/analysis, the investigators were unaware of sample identities and an independent experimenter handled all sample coding and decoding.

### 2.4 Behavioral assessments

All behavior experiments were conducted in a sound-isolated room. In each group, ten animals underwent the open field test, and the remaining animals were assessed using the hanging wire test.

#### 2.4.1 Open field test

To assess the spontaneous locomotor activity, a wooden square box with a polished black floor (16 equal squares; 100 × 100 × 40 cm) was used (Ahmed et al., 2016). Each rat was individually allowed to explore freely for 10 min. The overhead camera recorded different parameters including the distance traveled, average velocity, and immobility duration. The videos were analyzed using ANY-maze video tracking software (Stoelting Co., United States). Locomotor activity parameters were quantified using automated thresholds set within the software (immobility was defined as movement below 2 cm/s for ≥1 s). All analyses were conducted by an assessor blinded to group allocation.

#### 2.4.2 Hanging wire test

Rats were permitted to grip a steel wire with their forelimbs for 90 s. The wire was stretched horizontally at a height of 50 cm above a cushioned surface (Shalaby et al., 2018). The duration each rat held onto the wire was documented.

### 2.5 Biochemical and ELISA assay

All protocols were carried out according to the manufacturer’s pamphlets. Striatal total antioxidant capacity (TAC) and malondialdehyde (MDA) were assessed using commercial Biodiagnostic (Dokki, Egypt) colorimetric kit (TA 25 13 and MD 25 29, respectively). Striatal B-cell lymphoma-2 (Bcl-2; SL0108Ra), BDNF (SL0131Ra), and IL-1β (SL0402Ra) were assessed using Sunlong kits (Hangzhou, China). Striatal p53 (CSB-E08336r) and TNF-α (CSB- E11987r) were assessed using Cusabio kits (Wuhan, China).

### 2.6 Western blot

Briefly, striatal tissues were rinsed and homogenized in ice-cold lysis buffer containing protease and phosphatase inhibitor cocktails (Sigma, United States). The protein concentration was quantified colorimetrically. For immunoblotting, 30 µg of protein was incubated overnight at 4 °C with primary antibodies against: SIRT1 (1:1000; Cat. No. PA5-20964, Thermo Scientific, United States) and β-actin (1:1000; Cat. No. A5060, Sigma, United States). Membranes were washed and probed with horseradish peroxidase (HRP)-conjugated secondary antibodies (Dako, Denmark). Protein bands were detected using Western Lightning™ Plus ECL (Perkin Elmer, United States) and imaged with a ChemiDoc system (Bio-Rad, United States). Band intensities were normalized to β-actin and analyzed using Biorad ImageLab software. Means of bands optical densities were measured and their corresponding background subtracted, and then the subtracted intensities were divided on to their corresponding β-actin bands intensities (normalization), and the control group is set to “1.” (Burnette, 1981).

### 2.7 qRT-PCR

The expression levels of Nrf2, HO-1, and TLR4 were quantified by qRT-PCR following previously earlier reports (Sambrook et al., 1989; Burnette, 1981; Nasser et al., 2022). Briefly, total RNA was extracted using the SV Total RNA system (Cat. No. Z3100, Promega, Madison, WI, United States), and the purity was verified by the OD 260/280 nm absorbance ratios. Equal quantities of purified RNA were reverse transcribed to cDNA (Fermentas RT-PCR kit, Waltham, MA, United States). Quantitative PCR was performed using SYBR Green JumpStart Taq ReadyMix (Cat. No. S4438, Sigma–Aldrich, St. Louis, MO, United States). Primer specificity was confirmed by melt curve analysis showing a single sharp peak for each gene. Data were normalized to housekeeping genes and analyzed using the 2^−ΔΔCT^ method. Primer sequences are listed in Table 1.

**TABLE 1:** The sequence of all used primers.

Gene	Primer sequence	Accession number
Nrf2	F: 5′- GCCAGCTGAACTCCTTAGAC-3′ R: 5′- GATTCGTGCACAGCAGCA-3′	NM_031789.2
HO-1	F: 5′-CGACAGCATGTCCCAGGATT-3′ R: 5′-TCGCTCTATCTCCTCTTCCAGG-3.’	NM_012580.2
TLR4	F: 5′-CATGACATCCCTTATTCAACCAAG-3′ R: 5′-GCCATGCCTTGTCTTCAATTG-3′	NM_019178.2
β-actin	F: 5′- AGGGAAATCGTGCGTGACAT-3′ R: 5′- GAACCGCTCATTGCCGATAG-3′	NM_031144.3

### 2.8 Histopathology and Nissl staining for neuronal survival rate

Brain tissues were fixed in 10% neutral buffered formalin, dehydrated through a graded alcohol series, cleared in xylene, and embedded in paraffin wax. Five µm sections were cut and stained with hematoxylin and eosin (H&E) for histological examination by light microscopy. Nissl staining was used to evaluate the mean neuronal survival rate in each group (Gendy et al., 2023b).

### 2.9 Immunohistochemistry (IHC)

Brains tissue sections were mounted on adhesive slides, deparaffinized and re-hydrated to distilled water, subsequently a heat-induced epitope retrieval step was performed. Tissue sections were incubated for an hour at room temperature with primary anti-Caspase-3, anti-NF-қB (at a dilution of 1:200, Sanat Cruz, biotechnology, Inc.) and anti-GFAP (at a dilution of 1:300, Abbexa, United Kingdom). After washing, the HRP-labelled detection kit (BioSB, United States) was used to develop the color. Mayer’s hematoxylin was used as counter stain. Negative controls were processed without incubation with primary antibodies. Protein expression was quantified as mean area percentage in random non-overlapped five fields in each section using CellSens dimensions Olympus software (Olympus, Japan) (Gendy et al., 2023b).

### 2.10 Statistical analysis

Data were analyzed using GraphPad Prism 9.0.0 (United States) and expressed as mean ± standard deviation (SD). Data normality was assessed using the Shapiro–Wilk test and homogeneity of variance was evaluated using Levene’s test. Statistical comparisons were performed by one-way ANOVA with Tukey’s *post hoc* test. Statistical significance was set at p < 0.05.

## 3 Results

### 3.1 FA impact on the body weight

**FIGURE 2:**
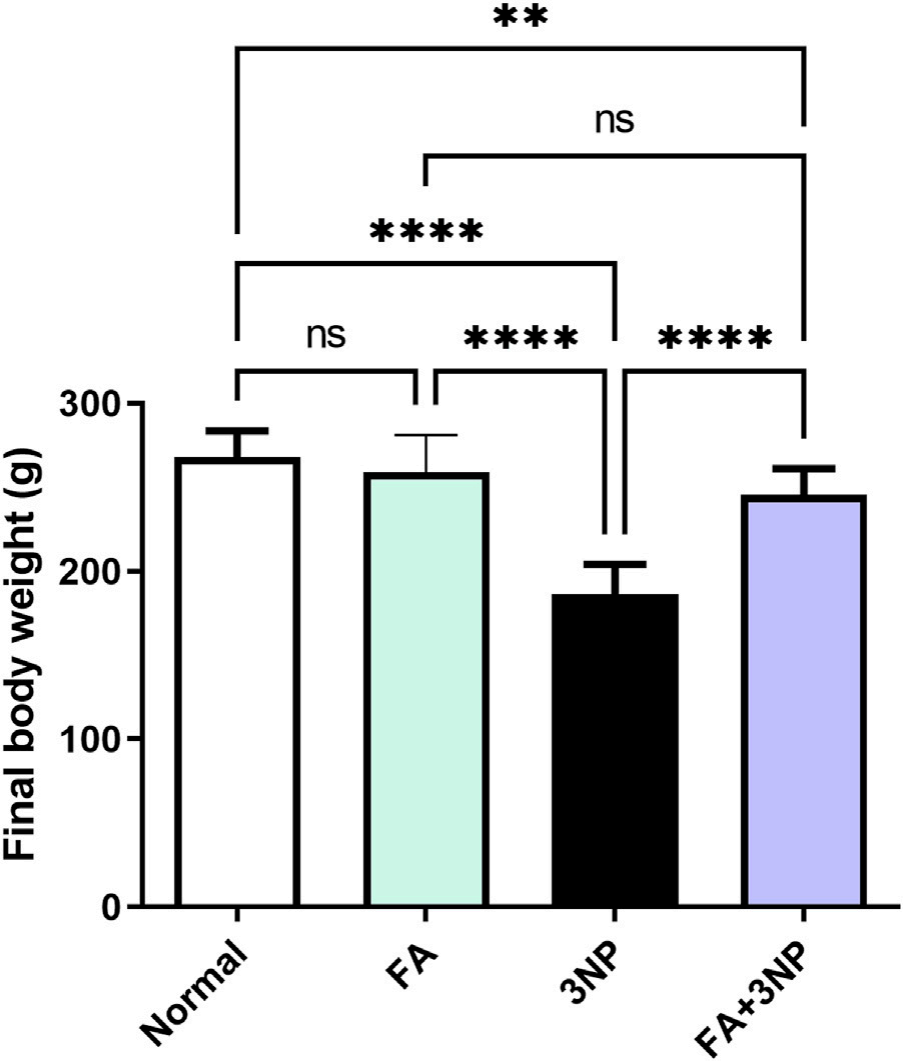
FA intake impacts on the final body weight in rats intoxicated with 3NP. Data are presented as mean ± SD; ns (non-significant, P > 0.05), *P < 0.05, **P < 0.01, ***P < 0.001, and ****P < 0.0001.

3NP administration induced significant body weight reduction (30% of normal group values; 186 ± 17.9 g vs. 268 ± 15.9 g in the normal group, p < 0.0001). Notably, the FA+3NP group showed significantly greater weight recovery (246 ± 15.5 g, p < 0.0001) compared to 3NP-treated animals (Figure 2).

### 3.2 FA impact on behavioral and motor studies

**FIGURE 3:**
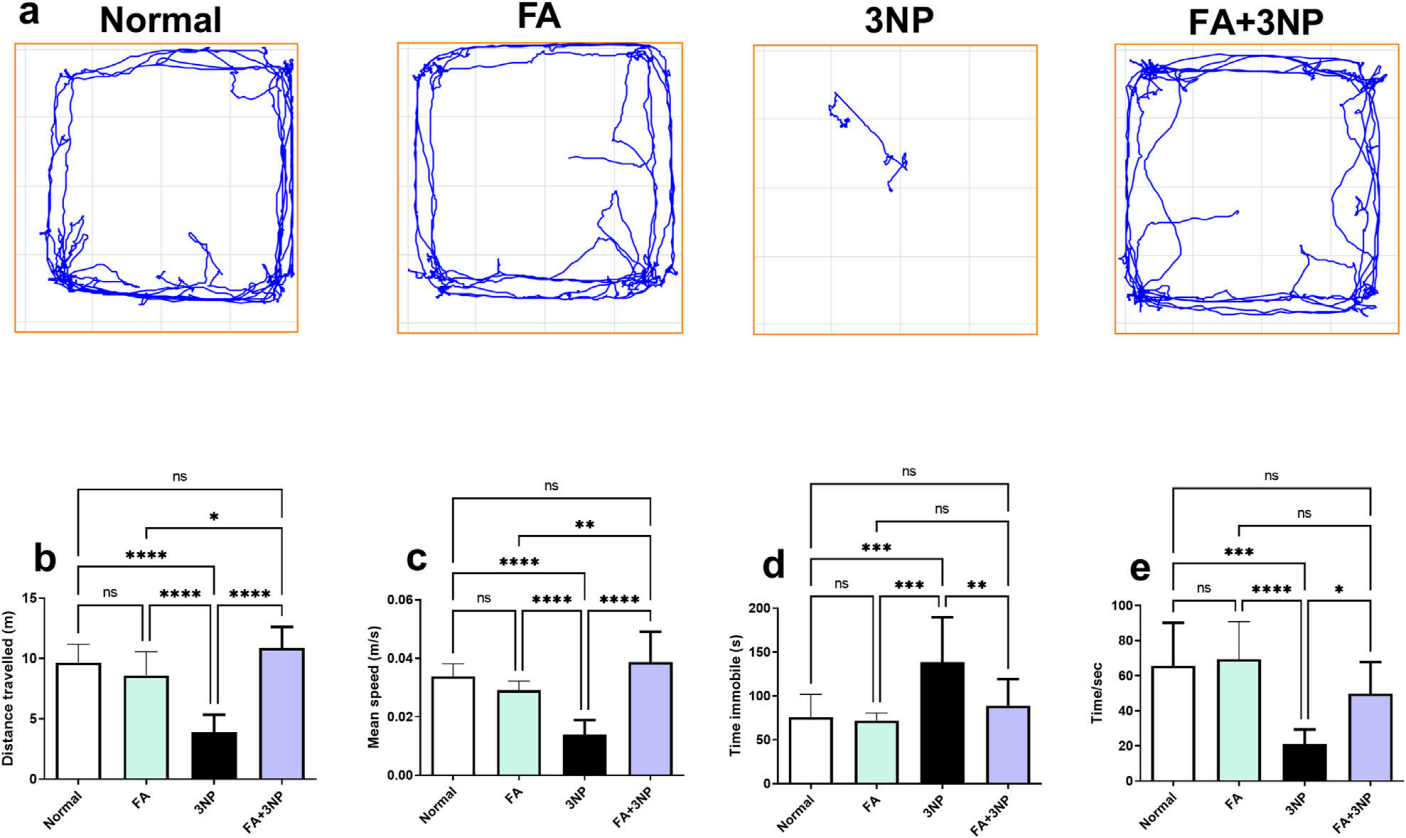
FA intake impacts on motor parameters **(a)**, representative movement tracks; **(b)**, distance traveled; **(c)**, speed average; **(d)**, immobility time and **(e)** fall of latency in rats intoxicated with 3NP. Data are presented as mean ± SD; ns (non-significant, P > 0.05), *P < 0.05, **P < 0.01, ***P < 0.001, and ****P < 0.0001.

As presented in Figure 3, 3NP intoxication induces striatal damage, resulting in locomotor impairment. The 3NP group exhibited significant behavioral impairments in the open field test including decreased total distance traveled (3.86 ± 1.48 m vs. 9.66 ± 1.52 m, p < 0.0001), reduced mean speed (0.0138 ± 0.005 m/s vs. 0.0338 ± 0.0043 m/s, p < 0.0001), and prolonged immobility time (139 ± 51.1 s vs. 75.7 ± 26.1 s, p < 0.001). Similarly, fall-off latency in the hanging wire test decreased to 21.1 ± 8.24 s vs. 65.6 ± 24.6 s in the normal group (p < 0.001). FA treatment significantly mitigated these locomotor deficits, increasing total distance traveled (10.9 ± 1.74 m, p < 0.0001 vs. 3NP), mean speed (0.0387 ± 0.0104 m/s, p < 0.0001), and fall-off latency (49.8 ± 18.0 s, p < 0.05), while reducing immobility time (88.9 ± 30.4 s, p < 0.01 vs. 3NP) (Figure 3).

### 3.3 FA impact on Nrf2, HO-1, TAC, and MDA

**FIGURE 4:**
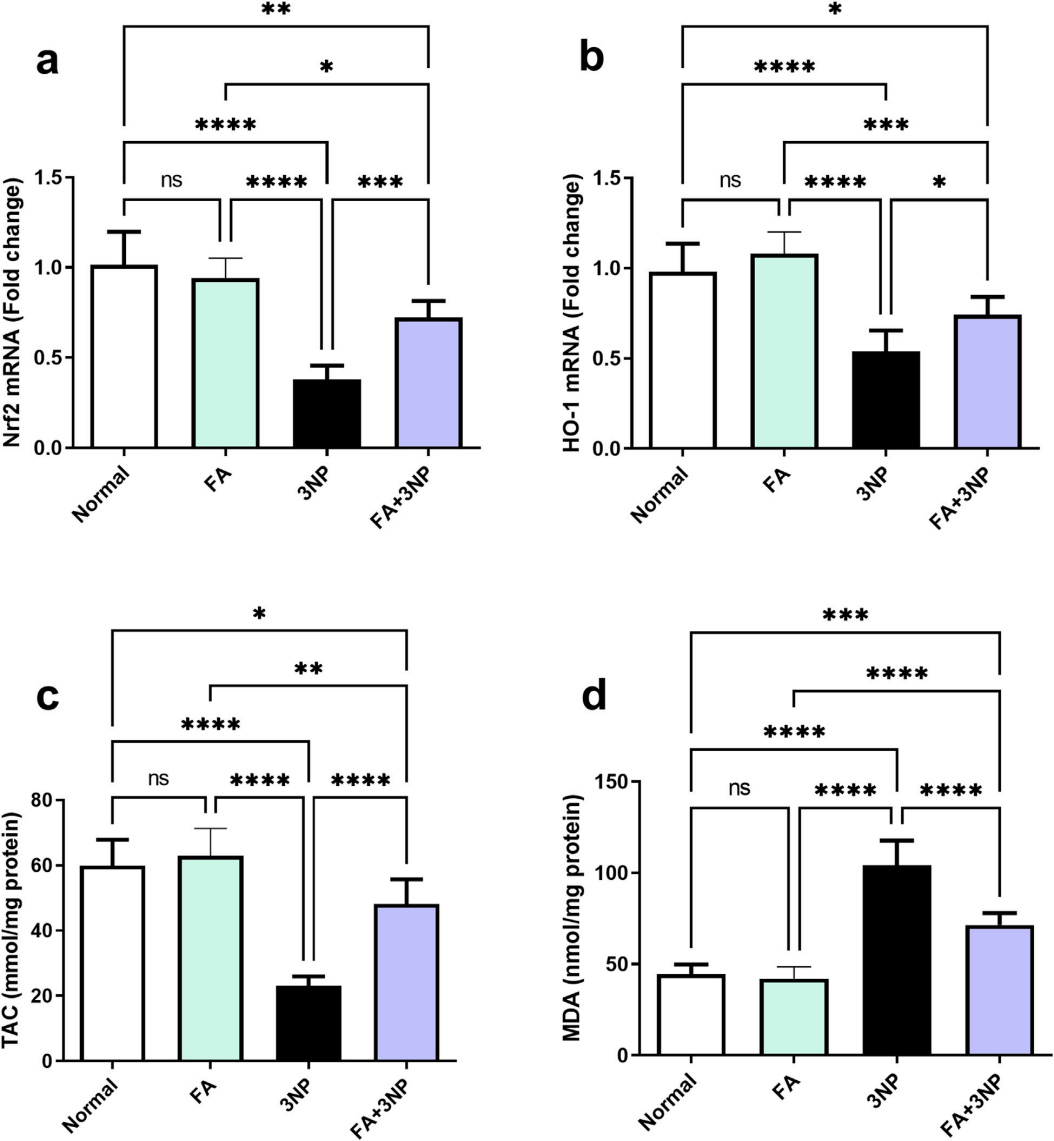
FA intake impacts on striatal **(a)** Nrf2 and **(b)** HO-1 mRNA expression, as well as **(c)** TAC and **(d)** MDA content in rats subjected to 3NP. Data are presented as mean ± SD; ns (non-significant, P > 0.05), *P < 0.05, **P < 0.01, ***P < 0.001, and ****P < 0.0001.

3NP administration significantly downregulated striatal Nrf2 mRNA expression (0.38-fold ±0.08 vs. 1.02-fold ±0.18 in the normal group, p < 0.0001) and HO-1 mRNA expression (0.54-fold ±0.12 vs. 0.98-fold ±0.16, p < 0.0001). Likewise, TAC levels were markedly reduced (23.1 ± 2.8 vs. 59.9 ± 7.9 mmol/mg protein, p < 0.0001), while MDA content was significantly elevated (104 ± 13.7 vs. 44.4 ± 5.4 nmol/mg protein, p < 0.0001) compared to the normal group. FA treatment significantly restored Nrf2 (0.72-fold ±0.09, p < 0.001) and HO-1 (0.74-fold ±0.10, p < 0.05) expression, enhanced TAC levels (48.2 ± 7.5, p < 0.0001), and decreased MDA (71.5 ± 6.5, p < 0.0001) compared to the 3NP group (Figure 4).

### 3.4 FA impact on BDNF

**FIGURE 5:**
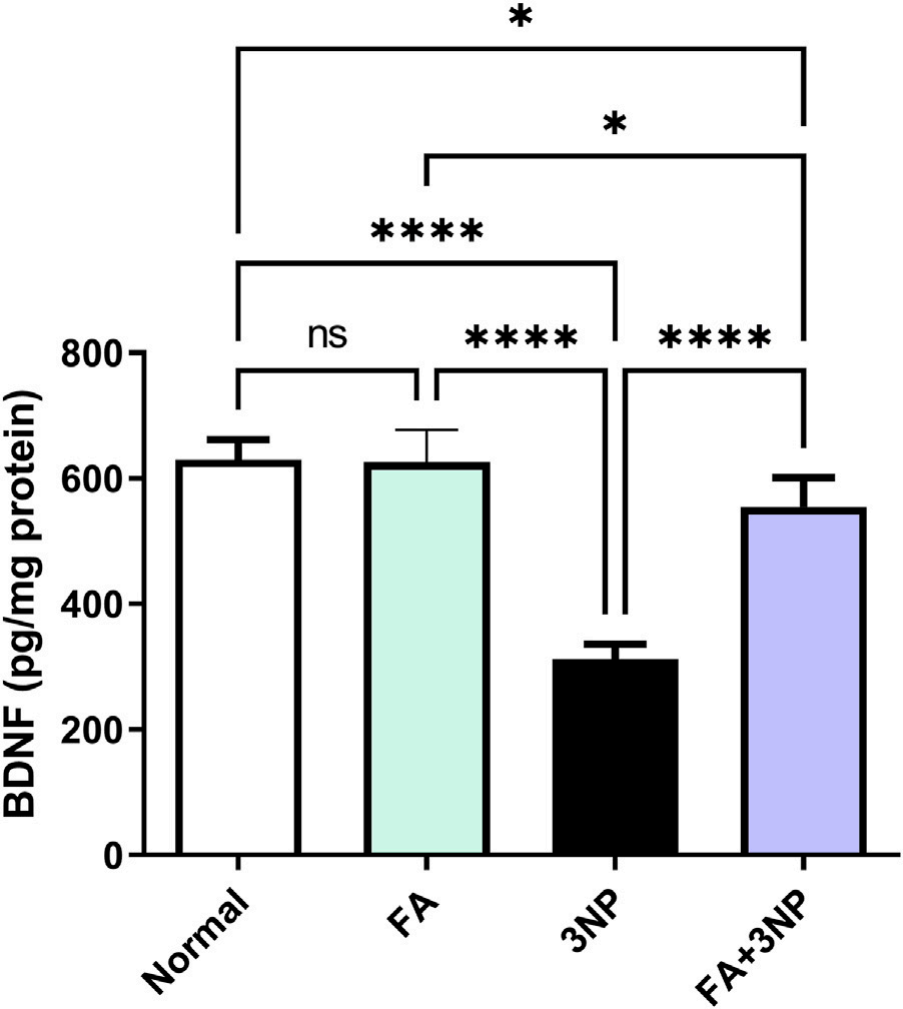
FA intake impacts on striatal neurotrophic BDNF content in rats subjected to 3NP. Data are presented as mean ± SD; ns (non-significant, P > 0.05), *P < 0.05, **P < 0.01, ***P < 0.001, and ****P < 0.0001.

As shown in Figure 5, intoxication by 3NP led to an approximate 50% reduction in striatal BDNF levels (Normal: 629 ± 32.2 pg/mg vs. 3NP: 312 ± 24.4 pg/mg, p < 0.0001). However, this decline was notably counteracted by FA treatment in the FA+3NP group (554 ± 47.1 pg/mg, p < 0.0001).

### 3.5 FA impact on TLR4, NF-κB p65, and inflammatory mediators

**FIGURE 6:**
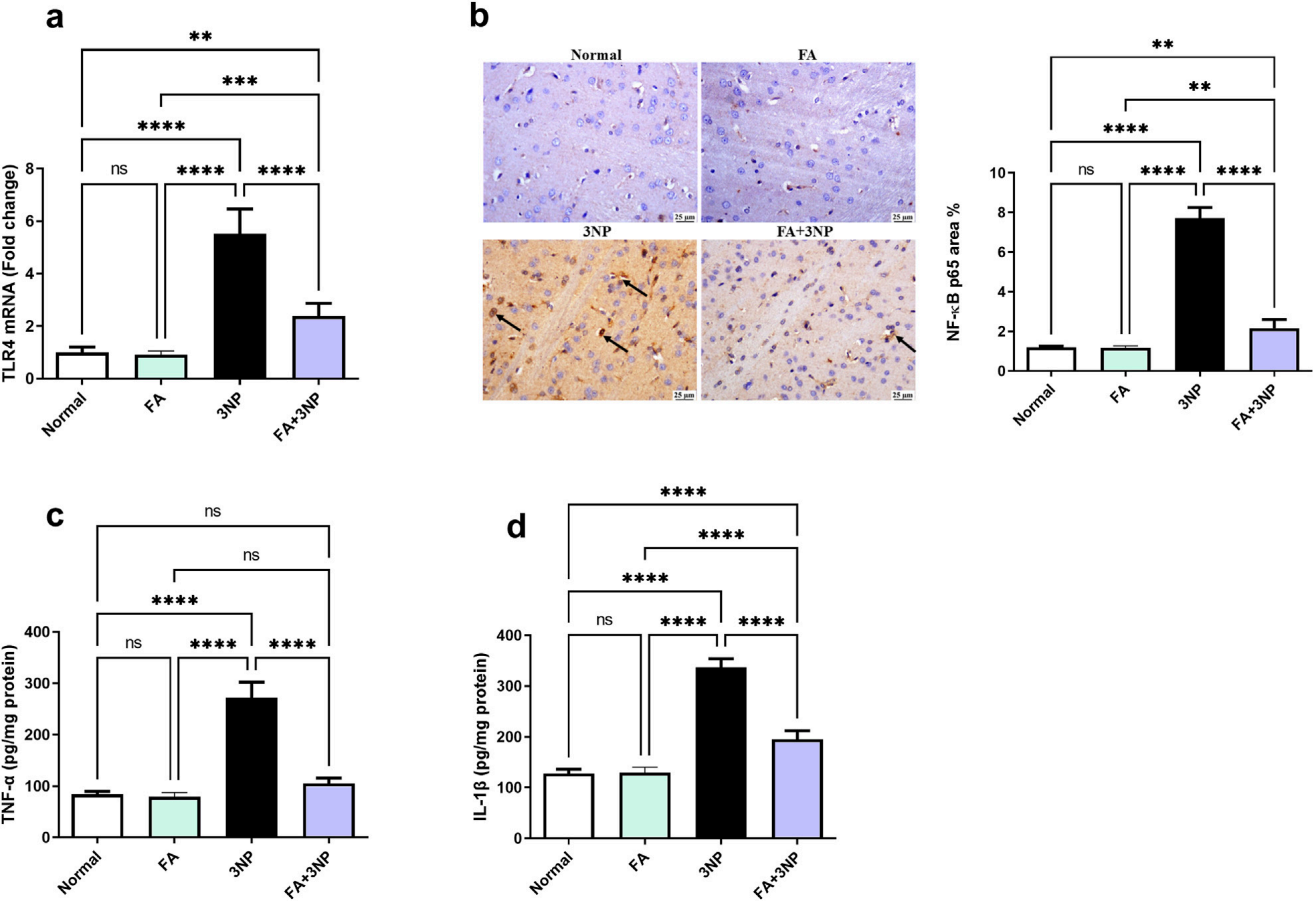
FA intake impacts on striatal **(a)** TLR4 mRNA expression, **(b)** NF-κB p65 IHC protein expression, as well as protein content of **(c)** TNF-α and **(d)** IL-1β in rats subjected to 3NP. Data are presented as mean ± SD; ns (non-significant, P > 0.05), *P < 0.05, **P < 0.01, ***P < 0.001, and ****P < 0.0001.

In contrast to the normal group (Figure 6), 3NP markedly enhanced TLR4 mRNA expression (5.5-fold ±0.9, p < 0.0001) and NF-κB p65 IHC protein content (7.7-fold ±0.5, p < 0.0001) leading to increment in TNF-α (Normal: 84.2 ± 5.74 pg/mg protein vs. 3NP: 272 ± 29.9 pg/mg protein, p < 0.0001) and IL-1β protein content (Normal: 128 ± 8.35 pg/mg protein vs. 3NP: 337 ± 16.9 pg/mg protein, p < 0.0001). Conversely, FA intake in 3NP-intoxicated rats counteracted these alterations certifying its anti-inflammatory effect (2.83-fold ±0.49, 2.16-fold ±0.43, 105 ± 10.3 pg/mg protein, and 195 ± 16.9 pg/mg protein, respectively).

### 3.6 FA impact on p53, Bcl-2, and caspase-3

**FIGURE 7:**
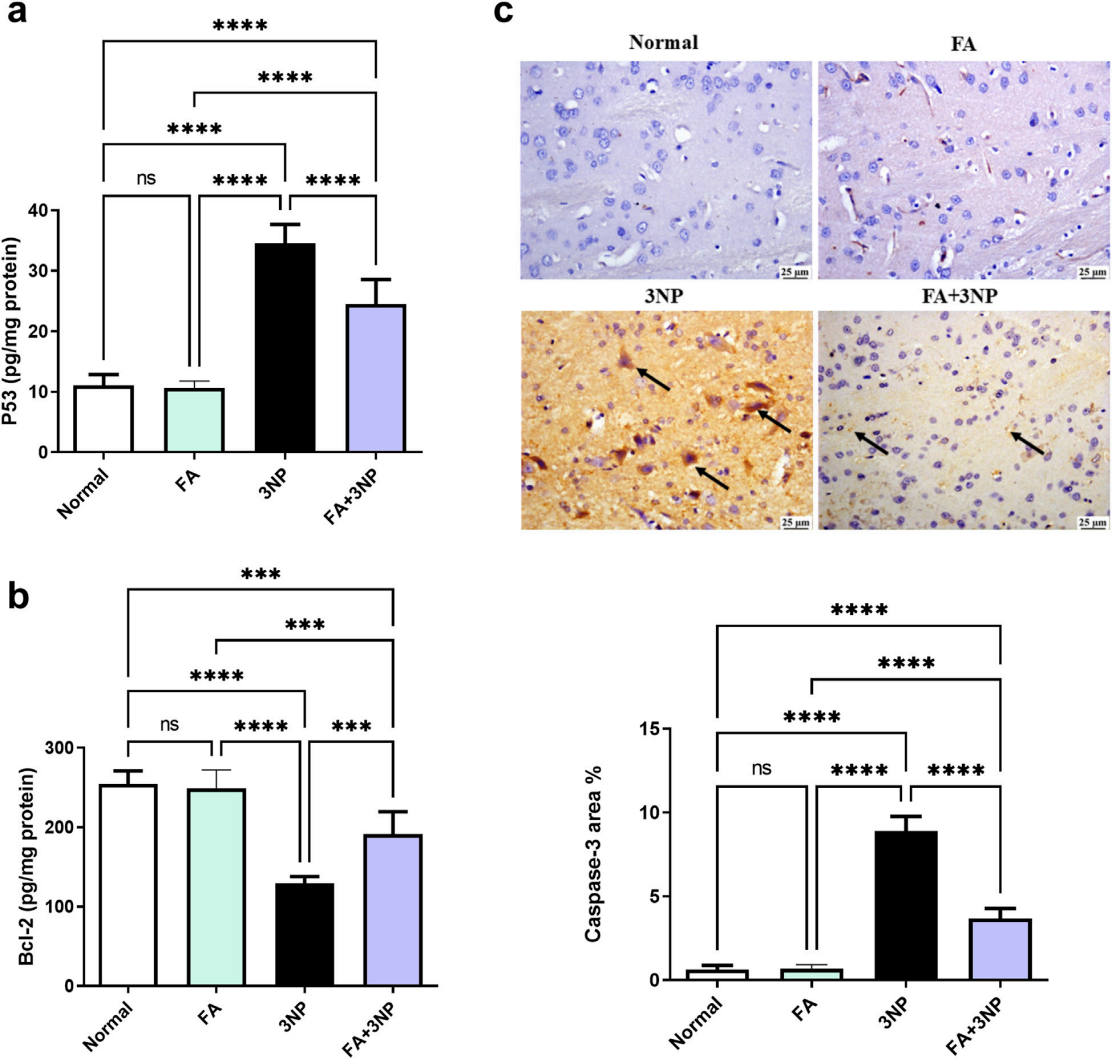
FA intake impacts on striatal **(a)** p53, **(b)** Bcl-2 content, and **(c)** caspase-3 IHC in rats subjected to 3NP. Data are presented as mean ± SD; ns (non-significant, P > 0.05), *P < 0.05, **P < 0.01, ***P < 0.001, and ****P < 0.0001.

The 3NP insult triggers apoptosis markers evidenced by a spike in both p53 content (34.5 ± 3.12 pg/mg protein, p < 0.0001) and caspase-3 IHC (8.91 ± 0.86-fold, p = < 0.0001) alongside reduction in Bcl-2 content (129 ± 8.68 pg/mg protein, p < 0.0001). Notably, FA demonstrated its anti-apoptotic potential by counteracting these changes (Figure 7).

### 3.7 FA impact on SIRT1 expression

**FIGURE 8:**
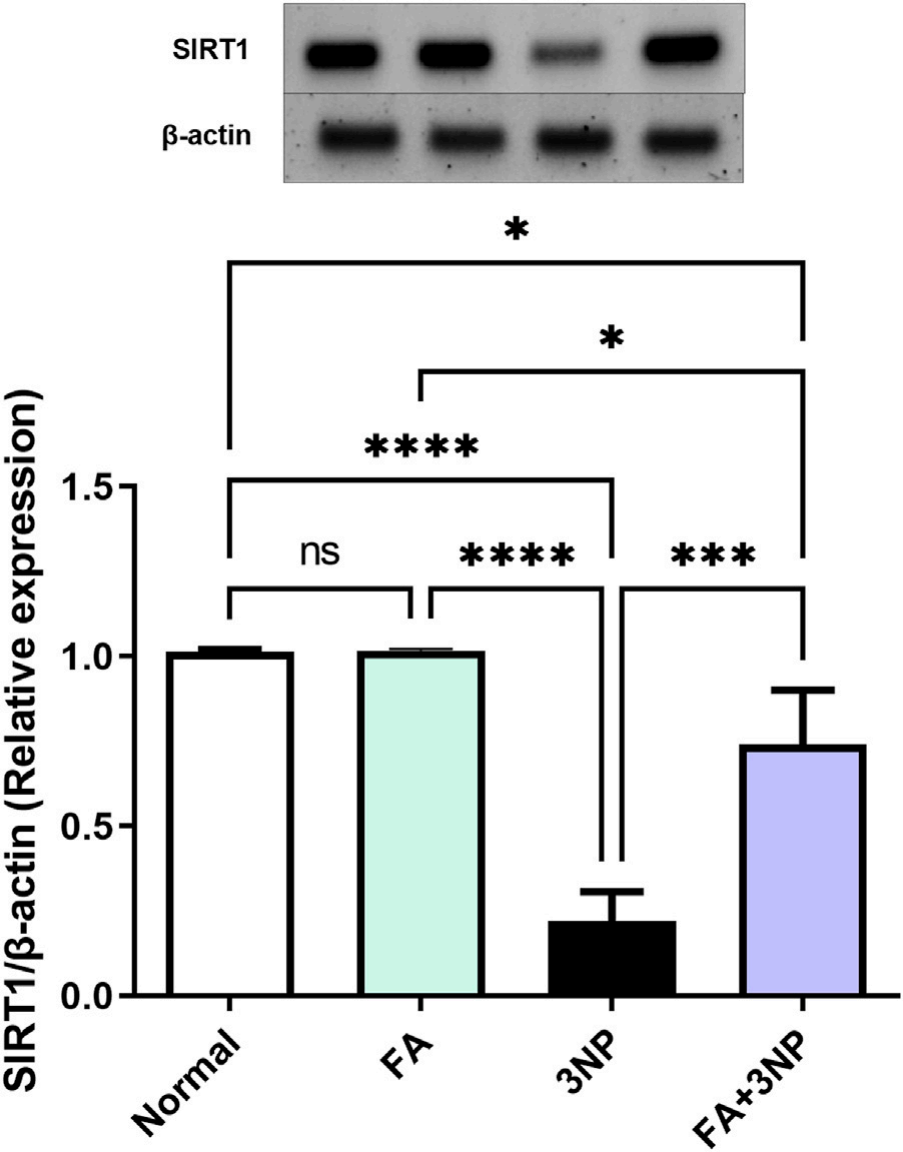
FA intake impacts on striatal protein expressions of SIRT1 in rats subjected to 3NP. Data are presented as mean ± SD; ns (non-significant, P > 0.05), *P < 0.05, **P < 0.01, ***P < 0.001, and ****P < 0.0001.

At the molecular level, 3NP disrupts cellular function by reducing SIRT1 protein expression by 21.7% (0.22-fold ±0.07, p < 0.0001). However, FA effectively counteracted this alteration (0.74-fold ±0.16, p < 0.001), demonstrating a protective regulatory role (Figure 8).

### 3.8 FA impact on the histopathological findings

**FIGURE 9:**
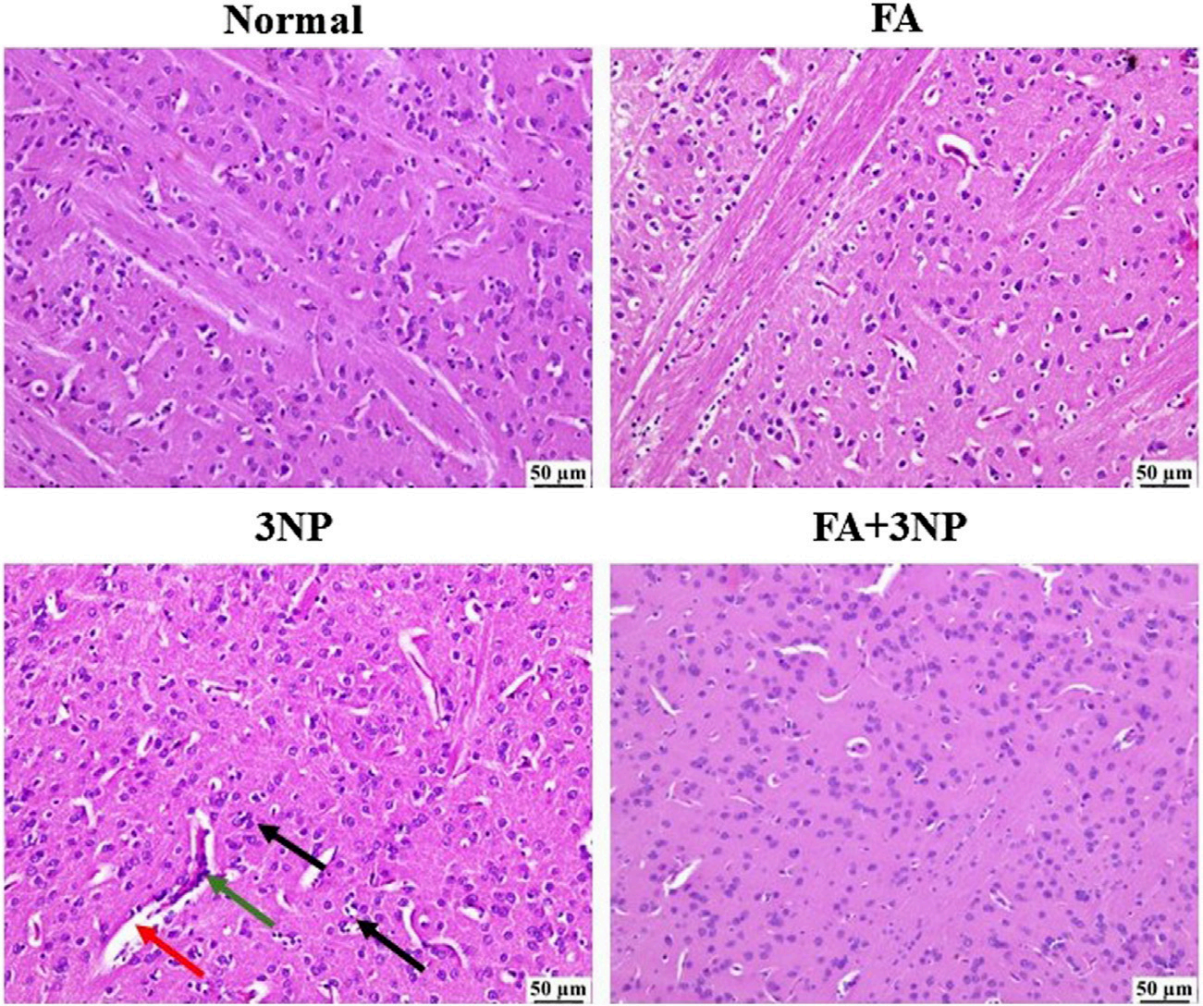
Representative photomicrographs of H & E stained stratia in the normal and FA groups showing intact neurons with normal histological architecture. 3NP group, showing gliosis (black arrow), distinct capillaries (green arrow), and edema (red arrow). FA+3NP group, showing apparently normal striatum.

As shown in Figure 9, microscopic evaluation of brain sections from the normal group revealed normal structure of striatum. Likewise, no histopathological changes were detected in the sections examined from the FA group. On the contrary, 3NP group showed focal gliosis with marked edema as well as the existence of some dark degenerating neurons within the striatum. Marked improvement was detected in the examined sections from FA+3NP group as sporadic focal gliosis was detected meanwhile apparently normal stratum was detected in almost all examined sections.

**FIGURE 10:**
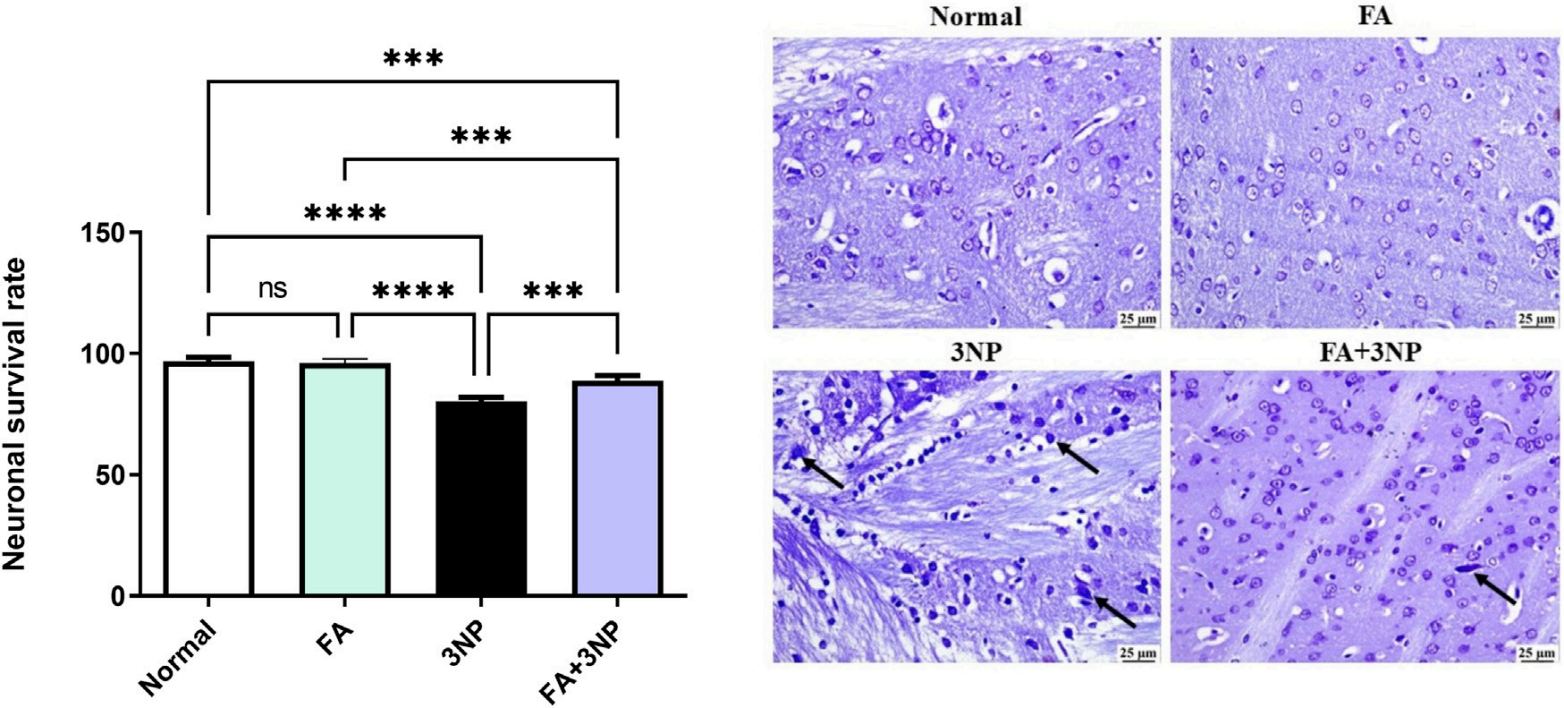
FA intake impacts on the neuronal survival rate in rats exposed to 3NP. Normal and FA groups, showing apparently normal lightly stained neurons within the striatum. 3NP group, showing numerous dark degenerating neurons (arrows) within the striatum. FA+3NP group, few dark degenerating neurons (arrows) within the striatum. Data are presented as mean ± SD; ns (non-significant, P > 0.05), *P < 0.05, **P < 0.01, ***P < 0.001, and ****P < 0.0001.

Moreover, as illustrated in Figure 10, neuronal survival rate was dramatically lowered in 3NP group when compared to the normal control group. FA+3NP group showed meaningful elevation in neuronal survival rate when compared to the 3NP group. No notable difference was observed between the normal and the FA group.

**FIGURE 11:**
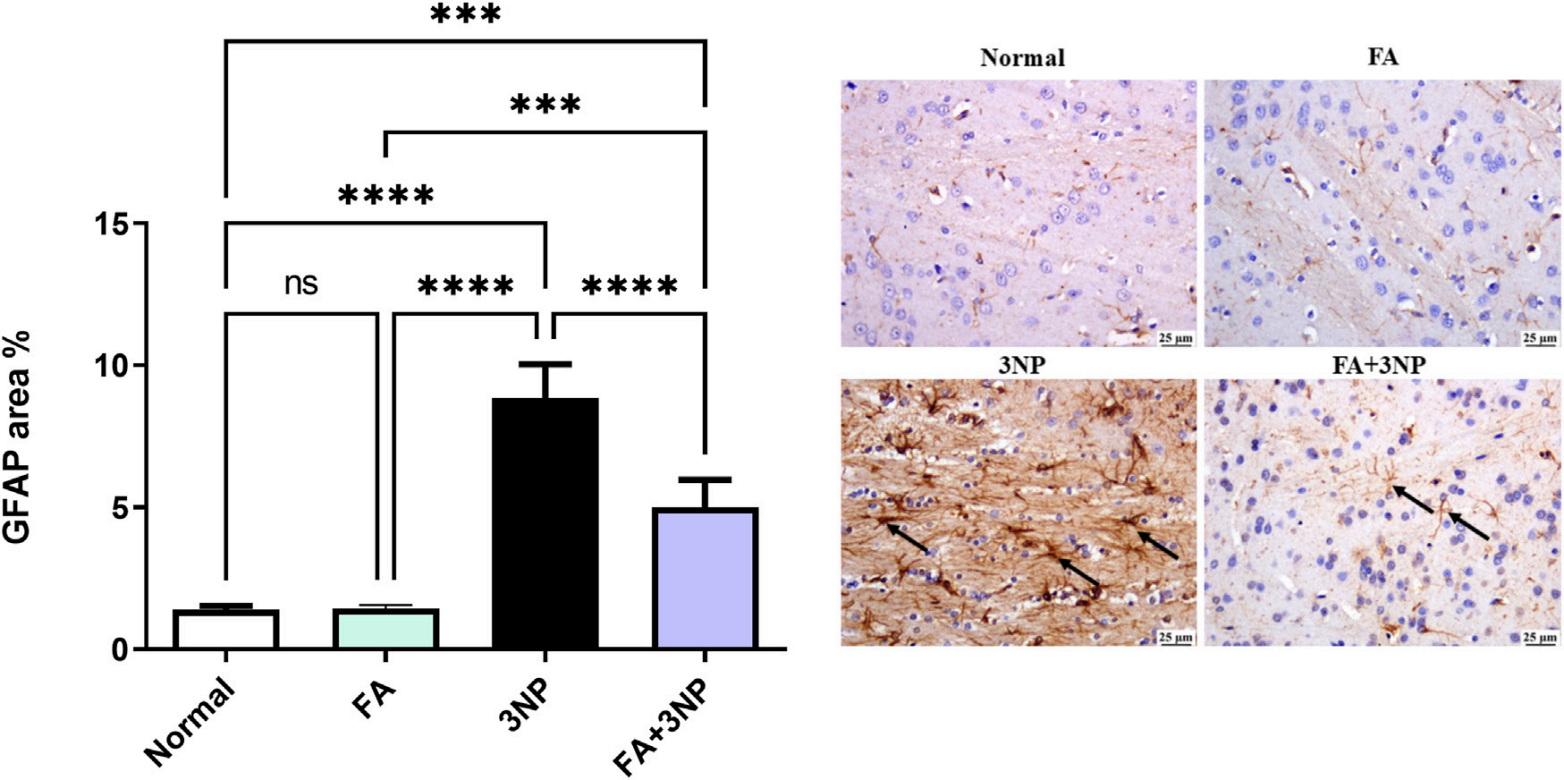
FA intake impacts on striatal GFAP in rats exposed to 3NP. Photomicrograph of brain, striatum, Normal and FA groups showing normal mild GFAP expression (Immune staining), 3NP group showing intense GFAP expression, and FA+3NP group showing moderate GFAP expression. Data are presented as mean ± SD; ns (non-significant, P > 0.05), *P < 0.05, **P < 0.01, ***P < 0.001, and ****P < 0.0001.

In addition, the 3NP group disclosed a marked upsurge in GFAP expression compared to the normal group. However, the FA+3NP group exhibited noteworthy shrinkage in GFAP expression relative to the 3NP group (Figure 11).

## 4 Discussion

In this study, we employed the established 3NP model to induce HD. This model allowed us to assess the potential neuroprotective effects of FA against multiple detrimental events associated with the disease. Our findings extend earlier reports of FA neuroprotection. Specifically, we provide possible mechanistic evidence that links FA’s behavioral and histological benefits to the simultaneous regulation of SIRT1, Nrf2, and NF-κB signaling in the 3NP-induced HD model. Unlike prior studies, which have not examined these pathways collectively in a single model, we demonstrate a new insight into the possible crosstalk that may underlie FA’s neuroprotective actions. These findings were further supported by results from qRT-PCR, Western blot, and immunohistochemistry. This data can position FA as a promising modulator of multiple pathogenic pathways in HD.

We investigated how this phenolic acid influenced body weight and motor behavioral parameters using open field and hanging wire tests. Additionally, we evaluated the antioxidant properties of FA by measuring levels of Nrf2, HO-1, TAC, and MDA. In terms of inflammation, we analyzed the cytokines TNF-α, and IL-1β. Additionally, the current study investigated the contribution of the TLR4/NF-κB p65 signaling in HD pathogenesis after FA treatment. Furthermore, we examined the apoptotic activity by assessing p53, Bcl-2, and caspase-3 levels. The expression of GFAP and BDNF was also evaluated alongside histopathological changes. Finally, the role of SIRT1 and its crosstalk with Nrf2, NF-κB, and p53 were discussed.

In the current research, the induction of HD by 3NP caused a decrease in the body weight of the animals, which aligns with findings from several previous studies (Elbaz et al., 2025; Mustafa et al., 2021). This weight loss may reflect the negative impact of 3NP on the animals’ energy metabolism, as well as motor deficit, anorexia, and the reduced food intake (Ahmed et al., 2016). Fortunately, FA intake was able to counteract these negative effects of 3NP, leading to a restoration of the rats’ body weight.

Alongside, significant changes in the neurobehavioral functions were observed during the open field tests. Following the administration of 3NP, animals exhibited a noticeable reduction in the total distance traveled and a decrease in mean speed, along with an increase in the time spent immobile. Conversely, treatment with FA effectively mitigated these issues, aligning with findings from earlier research (Zeni et al., 2012). The decline in muscle function, characterized by muscle weakness, is a primary indicator of HD and can be evaluated through grip strength tests, such as the hanging wire test. The test revealed significantly impaired limb strength following 3NP administration, as evidenced by reduced latency to fall. These findings are consistent with previous studies (Sayed et al., 2020; Elbaz et al., 2025). Conversely, administering FA led to an improvement in grip strength, as demonstrated by hanging duration enhancement. Overall, the neurobehavioral improvements associated with FA highlight its beneficial role in addressing the neurobehavioral abnormalities linked to HD.

Previous research has revealed that the neurotoxin (3NP) can induce oxidative stress in the striatum and other brain regions (Túnez et al., 2010; Sayed et al., 2020; Gao et al., 2015), where 3NP readily penetrates the blood-brain barrier and generate reactive oxygen species leads to pathological symptoms that resemble those associated with HD (Gonchar et al., 2021). Our study supports this finding as evidenced by the depletion of TAC following 3NP exposure, as well as increased levels of MDA which initiates lipid peroxidation. Similar outcomes have been documented in earlier studies (Mustafa et al., 2021; Gao et al., 2015; Sayed et al., 2020). The amplified oxidative state and neuronal damage resulting from 3NP injection may be partially due to the clear repression of the Nrf2/HO-1 antioxidant signaling as indicated herein. The Nrf2/HO-1 pathway is a well-known regulator of intracellular antioxidative processes. Its protective role in HD has been well documented (Sharma et al., 2025; Sethi et al., 2025). Nrf2 has been shown to promote the transcription of BDNF which is recognized for its critical functions in neuronal survival, neurogenesis, synaptic plasticity (Azman and Zakaria, 2025). On the other hand, BDNF is involved in the translocation and activation of Nrf2, which contributes to the restoration of redox homeostasis. It has been previously reported that levels of BDNF protein are diminished in animal models of HD. as documented herein and previously (Gendy et al., 2023b; Sayed et al., 2020). Moreover, HO-1 is known to provide neuroprotection against oxidative stress (Neis et al., 2018). In contrast, treatment with FA countered all markers of oxidative stress by restoring TAC levels while reducing MDA which can be attributed to the enhancement of Nrf2/HO-1 signaling (Mishra et al., 2022; Li et al., 2020b; Nouri et al., 2023). This indicates the antioxidant properties of FA in HD suggesting that antioxidants can modify or delay clinical manifestations including deficits in memory and motor skills associated with the disease (Sharma et al., 2021).

Another important signaling is TLR4-NF-κB p65 signaling where the current results indicated a rise in TLR4 content and NF-κB p65 immunoreactivity consistent with the findings of El-Abhar et al. (El-Abhar et al., 2018). TLR4 emerges as a significant molecular player in HD where its spike is known to contribute to the pathological status of multiple neurodegenerative disease (Dabi et al., 2023) as it plays an important role in the biochemical and neurological alterations (Dabi et al., 2023). Additionally, the pivotal role of NF-κB in the central nervous system via TLR4 overexpression has been well verified (Sharma et al., 2024), facts that coincide the current results. The high rate of TLR4 content can be linked to the increased cytokine levels and glial activation (Dabi et al., 2023). On the other hand, the documented reduction in Nrf2 levels following 3NP administration plays a role in enhancing NF-κB p65 levels, as Nrf2 is known to suppress NF-κB p65 subunit and its downstream inflammatory molecules (Ibrahim and Abdel Rasheed, 2022). FA depicted a decline in TLR4 content to concur with the results in LPS-induced neurotoxicity and sciatica models (Rehman et al., 2019; Zhang et al., 2023). Moreover, NF-κB p65 IHC observed attenuation was clear after FA intake which may be an outcome of decreased TLR4 levels. This hypothesis is evidenced by experimental data unveiling the role of FA-dependent downregulation in TLR4 for hindering NF-κB p65 and consequently serves as a key mechanism underlying FA anti-inflammatory effects. In this milieu, NF-κB inhibition may also result from Nrf2 activation by FA that has previously been documented to diminish NF-κB inflammatory character (Liu et al., 2021; Singh et al., 2022).

Inflammation also plays a significant role in HD pathogenesis (Valadão et al., 2020), as evidenced in the current study by the 3NP-induced increase in TNF-α and IL-1β levels, as well as the histopathological findings. These results align with a previous study investigating Dapagliflozin in the 3NP rat model of HD (El-Sahar et al., 2020). FA effectively mitigated inflammation by normalizing disrupted inflammatory parameters and enhancing histopathological outcomes. Similarly, FA has demonstrated anti-inflammatory properties in chronic unpredictable mild stress (Liu et al., 2017). Thus, attenuation of redox status and apoptosis via enhanced Nrf2 and reduced NF-κB p65 represents a potential mechanism underlying FA anti-inflammatory effects (Singh et al., 2022; Sharma et al., 2025; Gendy et al., 2023a).

The apoptotic death contributes a pivotal part in the neuronal degeneration in HD (Fan et al., 2017). In the present study, striatal exposure to 3NP elicited marked pro-apoptotic changes. This was evidenced by increased p53 content, upregulated caspase-3 immunoreactivity, and reduced levels of the anti-apoptotic protein Bcl-2. A result that match previous reports (Gendy et al., 2023a; Ibrahim and Abdel Rasheed, 2022; Ahmed et al., 2016). Importantly, our findings revealed that treatment with FA reversed these alterations by enhancing Nrf2 expression and reducing NF-κB p65 levels. Restoration of Nrf2 likely contributed to the reestablishment of redox homeostasis and upregulation of Bcl-2 (Singh et al., 2022), while suppression of NF-κB attenuated pro-apoptotic signals leading to reduced caspase-3 activation (Van Antwerp et al., 1998; Liu et al., 2021).

SIRT1 is an NAD-dependent enzyme plays a key role in the modulating of oxidative stress balance, inflammatory response, and apoptosis (Ren et al., 2019). Moreover, its role in HD is well documented (Naia and Rego, 2015; Jiang et al., 2012; Manjula et al., 2021). SIRT1 regulatory effect on apoptosis is well documented by inhibiting p53, leading to the downregulation of programmed cell death, as documented herein (Jazvinšćak Jembrek et al., 2021). Moreover, SIRT1 can restrain oxidative stress by activating Nrf2 nuclear translocation consequently amplifying transcriptional activation of antioxidant genes (Mosaoa et al., 2024; Sethi et al., 2025; Ibrahim and Abdel Rasheed, 2022). SIRT1 influences the interaction between Nrf2 and NF-κB. It promotes the expression of antioxidant genes expression while limiting pro-inflammatory cytokines production in response to oxidative stress (Jazvinšćak Jembrek et al., 2021). Additionally, SIRT1 plays a regulatory role in inflammatory factors transcription, including NF-κB, which is a crucial regulator of various pro-inflammatory cytokines (Peixoto et al., 2017; Song et al., 2022). Additionally, SIRT1 can influence neuroinflammation by decreasing the activation of astrocytes, which leads to a reduction in GFAP levels, a well-known marker associated with astrogliosis (Shaheen et al., 2021; Vaziri et al., 2001). Furthermore, SIRT1 has been shown to inhibit the activation of microglia and its detrimental inflammatory cascade (Li et al., 2020a). On the other hand, SIRT1 overexpression has been linked to BDNF expression (Harrison, 2012). Our findings verified the clear repression of SIRT1 after 3NP intoxication, while FA treatment reactivated this signaling (Chen et al., 2019; Wang et al., 2023). Based on these outcomes, it is reasonable to propose that the upregulation of SIRT1 by FA represents a potential mechanism underlying its therapeutic effects against 3NP-induced HD.

In the current research, the neurotoxicity induced by 3NP was associated with the activation of microglial cells, which was evidenced by the activation of astrocytes and reflected in the overexpression of GFAP. The administration of FA effectively protected the animals from astrocyte activation by lowering GFAP expression. This finding is consistent with previous study highlighting the defensive actions of FA in rats model of Alzheimer’s disease (Khalifa et al., 2025).

BDNF is recognized for its critical functions in stimulating neurogenesis, enhancing synaptic plasticity, promoting neuronal survival, and mitigating neuroinflammation caused by TNF-α (Patil et al., 2014). In HD, there is a notable decrease in BDNF expression (Pineda et al., 2005). Likewise, the 3NP model of HD has shown a significant reduction in BDNF levels, as previously reported (Sayed et al., 2020). One potential reason for this decline is the activation of NF-κB p65 induced by 3NP, which has been suggested in studies evaluating memory loss triggered by lipopolysaccharide (Gu et al., 2015). Fortunately, the administration of FA has been effective in preventing this reduction and reversing the neuronal damage caused by 3NP, a beneficial effect that has been documented previously in depressive-like behaviour model (Mallik et al., 2023). The beneficial effects of FA on BDNF restoration can also be linked to its modulation of SIRT1 and Nrf2 signaling. SIRT1 has been reported to enhance BDNF transcription and promote neuronal survival (Tang et al., 2020). Nrf2 activation can also stimulate BDNF expression as part of its neuroprotective effect (Cao et al., 2022). In our study, FA administration concurrently elevated SIRT1, Nrf2, and BDNF levels, indicating that the recovery of striatal BDNF may be mediated, at least in part, through these upstream regulators.

Neuronal damage was assessed using Nissl staining, which revealed a marked increase in the number of degenerated cells following the intraperitoneal injection of 3NP, as previously documented (Sayed et al., 2020; Ibrahim and Abdel Rasheed, 2022). Notably, FA intake demonstrated a neuroprotective effect by increasing neuronal survival. Finally, this study offers new insights into the neuroprotective effects of FA in the case of HD. The protective effect of FA is accompanied by changes in SIRT1/Nrf2/NF-кB/p53 signaling. These changes enhance the antioxidant capacity of striatal tissue while reducing both inflammatory and apoptotic responses.

Limitation of the current study: The use of different methodologies for apoptotic markers: ELISA for p53 and Bcl-2, and immunohistochemistry for caspase-3. This heterogeneity may affect direct comparability. The baseline behavioral performance was not assessed before 3NP administration. Future studies including baseline measurements will provide more assurance that the observed differences are due to treatment effects only. Moreover, the present study did not directly assess SIRT1 activity or p53 acetylation status. Likewise, we did not include pharmacological modulation of SIRT1 (e.g., inhibitor or activator) to establish causality. These experiments would allow confirmation that the observed molecular changes are specifically mediated via SIRT1 activation rather than parallel pathways. Future work will incorporate such approaches. Nonetheless, our findings are consistent with prior reports in neurodegenerative models showing that SIRT1 activation attenuates oxidative stress, inflammation, and apoptosis through these pathways, supporting the plausibility of our proposed mechanism. In addition, we acknowledge that a smaller sample size in the Western blot may limit sensitivity to detect more subtle protein changes. On the other hand, the observed effect sizes in our data were large enough to yield statistically significant difference. Future experiments will incorporate *a priori* power calculations to ensure that sample sizes are optimized prospectively for all endpoints.

## Data Availability

The datasets presented in this study can be found in online repositories. The names of the repository/repositories and accession number(s) can be found in the article/supplementary material.
